# Behavioral Changes and Hippocampus Glucose Metabolism in APP/PS1 Transgenic Mice via Electro-acupuncture at Governor Vessel Acupoints

**DOI:** 10.3389/fnagi.2017.00005

**Published:** 2017-01-24

**Authors:** Jin Cao, Yinshan Tang, Yujie Li, Kai Gao, Xudong Shi, Zhigang Li

**Affiliations:** ^1^School of Acupuncture Moxibustion and Tuina, Beijing University of Chinese MedicineBeijing, China; ^2^Department of Rehabilitation in Traditional Chinese Medicine, The Second Affiliated Hospital of Zhejiang University School of MedicineHangzhou, China; ^3^Institute of Medical Laboratory Animal Science, Chinese Academy of Medical SciencesBeijing, China

**Keywords:** electro-acupuncture, Alzheimer’s disease, micro-PET, glucose metabolism, hippocampus

## Abstract

**Objective:** Investigating the effects of electro-acupuncture (EA) treatment on mice with Alzheimer’s disease (AD), using Morris water maze (MWM) for spatial learning and memory behavior tests combined with micro-positron emission tomography (micro-PET) imaging for glucose metabolism in hippocampus.

**Methods:** Thirty seven-month-old APP/PS1 mice were randomly divided into AD Model group (AD group), medicine group (M group) and EA group, C57BL/6 mice were used for Normal control group (N group), *n* = 10 in each group. Mice in M group received donepezil intervention by gavage with dose at 0.92 mg/kg. EA was applied at Baihui (GV20) and Yintang (GV29) acupoints for 20 min then pricked at Shuigou (GV26) acupoint, while mice in N, M and AD groups were received restriction for 20 min, with all treatment administrated once a day for 15 consecutive days. After the treatment, MWM was performed to observe behavioral changes in mice, then hippocampus glucose metabolism level was tested by micro-PET imaging.

**Results:** Compared with that of AD group, the escape latency of M and EA groups declined significantly (*P* < 0.01), while the proportion of the platform quadrant swimming distance in total swimming distance showed an obvious increase (*P* < 0.01), and EA group occupied a higher percentage than that in M group. The micro-PET imaging showed that mice in AD group performed a lower glucose metabolic rate in hippocampus compared with N group (*P* < 0.01). Both M and EA groups presented a significant higher injected dose compared with AD group (*P* < 0.01), and the uptake rate of EA group was higher than M group.

**Conclusion:** Both donepezil and EA have therapeutic effects on AD mice. To a certain extent, EA shows a better efficacy in treatment of AD by improving the spatial learning and memory ability, while also enhancing glucose metabolism in hippocampus.

## Introduction

Alzheimer’s disease (AD), also called senile dementia, is a common central nervous system (CNS) degenerative disease, which is characterized by progressive memory impairments, aphasia, agnosia, executive dysfunction, accompanied with varying degrees of personality and behavior changes. With the advent of global aging society, AD has becoming a serious threat to humanity. Consequently, the prevention and treatment of AD has become an urgent issue that needs earnest study and solution.

Electro-acupuncture (EA), combining traditional acupuncture with modern electrotherapy, is a promising therapy that has been widely used in clinical practice, especially exerting beneficial effects in CNS diseases such as depression, spinal cord injury (SCI), stroke and AD (Guo et al., 2014; Mo et al., 2014; Deng et al., 2016; Lai et al., 2016). The mechanism of AD mainly lies in two aspects of deficiency in origin and enrichment in symptom in accordance with the Traditional Chinese Medicine (TCM) theory. Deficiency of essence and debility of brain marrow were the basic pathogenesis of AD, while stagnation of phlegm in brain collateral was reflected as an excessive symptom in superficial. There exists some indivisible relations between Governor Vessel (GV) and brain, both in distribution and in function. Clinical and experimental studies have shown that acupuncture at GV possessed therapeutic effects in AD treatment (Yang et al., 2013; Jiang et al., 2015; Wang et al., 2015).

Pathologically, AD is generally characterized by the synergy between the extracellular accumulation of Aβ plaques and presence of neurofibrillary tangles containing hyper-phosphorylated tau, which activates microglia and astrocytes then subsequent inflammatory reaction. A progressive damage of synapse leads to neuronal apoptosis, which consequently presents a glucose metabolism level decrease in specific regions of the brain (Brun and Englund, 1986; Grundke-Iqbal et al., 1986). Present studies on AD animal models and curative effect evaluation are mainly based on behavioral observation, neuro-biochemical and pathological tests. There is, however, some valuable imaging methods like magnetic resonance imaging (MRI), Position emission computed tomography (PET) and multiple modality imaging which can reflect objective and reliable brain structure and activity changes were often neglected.

In this study, we selected 7-month old APP/PS1 double transgenic mice as the AD animal model, and applied Morris water maze (MWM) and non-invasive *in vivo* imaging micro-positron emission tomography (micro-PET) to evaluate the effect of EA treatment. We preferred the GV acupoints Baihui (GV20), Yingtang (GV29) and Shuigou (GV26) as acupuncture prescription. Escape latency and target distance proportion were used to analyze spatial learning and memory ability, 18F-fluorodeoxy glucose (18F-FDG) uptake rate in each gram of hippocampus tissue presenting glucose metabolism levels. This study aims to evaluate the curable effect of EA on AD model mice, explore its mechanism and provide reliable experimental basis for clinical treatment of AD as expected.

## Materials and Methods

### Experimental Animals and Grouping

A total of 30, 7-month old, clean grade, male APPswe/PS1dE9 mice on a C57BL/6 background [B6/J-Tg (APPswe, PSEN1dE9) 85Dbo/Nju, ID: D000268, were randomly divided into AD model group (AD group), Medicine group (M group) and EA group, *n* = 10 in each group, with 10 aged-matched littermate negative transgenic mice served as normal control group (N group). All animals were sourced from Model Animal Research Center of Nanjing University [Certificate number: SCXK (SU) 2010-0001], with weight ranging in (30 ± 2) g. Laboratory animal barrier system is provided by Experimental Animal Centre of First Teaching Hospital of Beijing University of Chinese Medicine. Housed mice separately in single cages in a temperature-controlled room (23 ± 1°C), under 12-h light/dark cycle and (55 ± 5) % humidity, in avoidance of outside interference and fed ad-libitum on standard chow with open access to water. All experimental procedures were in accordance with the Guidelines for the Care and Use of Laboratory Animals of the Ministry of Science and Technology of the People’s Republic of China, and the Experimental Animal Research Ethics Committee of Beijing University of Chinese Medicine approved the study protocol. We made all efforts to minimize animal suffering and sacrifice in this experiment process.

### Reagents and Apparatus

Reagents and apparatus are displayed in **Table 1**.

**Table 1:** Reagents and apparatus used in research.

Reagents and apparatus	Source	Details
Donepezil Hydrochloride Tablets	eisai (China) Co., Ltd	Certificate number: H20050978, specification: 5 mg per pill × 10 pills
Disposable sterile acupuncture needle	Beijing Zhongyan Taihe Medical Instrument, Co., Ltd	Model: ZYTH2013030504, specification: 0.25 mm × 13 mm
Electro-acupuncture (EA) apparatus	Peking University Institute of Science Nerve and Beijing Hua Wei Industrial Development Company	Model: HANS-LH202
18F-FDG tracer	PET-CT Center, Cancer Hospital, Chinese Academy of Medical Sciences, Peking Union Medical College	—
Inveon Micro PET/CT scanner	Siemens Preclinical Solution USA, Inc., Knoxville, TN, USA	Provided by the Institute of Laboratory Animal Science, Chinese Academy of Medical Sciences and Comparative Medical Center, Peking Union Medical College
Morris water maze	Shanghai Xinruan Information Technology, Co., Ltd	Model: XR-XM101
Software of image acquisition and analyzing system	China Daheng Group, Inc.	China Daheng Group, Inc., Beijing Image Vision Technology Branch

### Methods of Treatment

Mice in N group received no treatment, but were grabbed and bound when mice in other groups were receiving treatments to ensure an equivalent condition. Mice in AD group were treated in accordance with N group. Donepezil hydrochloride tablets were crushed and dissolved in distilled water, with the dose at 0.92 mg/kg and delivered to mice by oral administration in M group, which were grabbed and bound as N and AD group.

**FIGURE 1:**
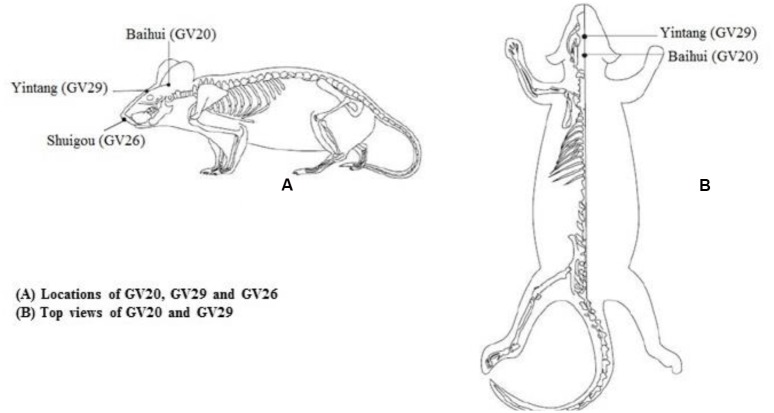
**Mouse acupoints location we applied in this study**.

Mice in EA group received EA treatment. The acupoints Baihui (GV20), Yintang (GV29) and Shuigou (GV26) in GV were chosen according to document (Li, 2007; Guo and Fang, 2012). Acupoints location in mouse were well-correspond to the anatomical site of human acupoints, GV20 is at the midpoint between the auricular apices, GV29 is located midway between the medial ends of the two eyebrows, while GV26 is located below the mouse nasal septum, at the junction of the lower two thirds and upper one third of the cleft lip midline (**Figure 1**). Sterile acupuncture needles were separately inserted with downward horizontal at GV20 and upward horizontal at GV29, 0.5 cm in depth. Then needles were connected to a programmed apparatus, and stimulated with sparse waves (2 Hz in frequency, 2 V in voltage, and 0.1 mA in electric current) generated from EA apparatus, which would be the most preferable if slight trembling of animal’s head was observed. Vertical pricking at GV26 acupoint was manipulated at the end of EA treatment. All treatment approaches should be implemented for 20 min, once a day for 15 consecutive days.

### Morris Water Maze Test

Morris water maze (MWM) is a well-validated test for spatial learning and memory. Over the course of test, the experiment room should maintain a sound insulation, indirect light source and low-light situation environment, with remaining objects in this room at the original location and experimental conditions should be unchanged. After previous treatments, mice were tested in a circular water tank (90 cm in diameter and 50 cm in depth), which was randomly divided into four equal quadrants (I, II, III, IV) and set an escape platform (circular, 5 cm in diameter and 28 cm in height) at the center of III quadrant. The tank was filled with warm water (22 ± 2°C) and made opaque by adding 0.5 kg skim milk powder to the depth of 30 cm. Visual cues in different shapes were placed on tank wall of each quadrant, in plain sight of the mice. All trials were tracked automatically by a digital camera (set at 2 m ceiling straight above the water tank center) and connected with image acquisition system. With the aim to evaluate the changes of learning and memory ability, each animal received a 5-day hidden platform trial then a 1-day probe trial. In order to familiarize mice with the test environment, experimental animal freely swam in the water tank (without platform) for 90 s in the morning and afternoon separately on the day before the hidden platform trial. Mice were placed the on the platform for 10 s to observe the surrounding environment first, and the hidden platform remained in a fixed location throughout the trail. Then, we selected three entry points in I, II, IV quadrant respectively, which should have equal distance to the center of the tank, and experimenter gently placed the animal into the water with its head toward the tank wall. We recorded the escape latency (expressed by the swimming time spent on finding submerged platform) and the swimming route if animal found the hidden platform within 60 s and remained on it for 5 s. If animal could not find the platform in 60 s, it would be removed from the water and placed on the platform for 10 s to observe and memorize the location, then we recorded 60 s as its escape latency. The hidden platform trial repeated for five consecutive days. For the probe trial, removed hidden platform and placed mice in I, II, IV quadrant respectively, recorded whole swimming route and time spent on III quadrant within 60 s, to analyze their spatial memory and learning ability. During the trials, all mice were placed back in their resident cages using a spoon-net in order to avoid direct contact with the experimenter.

### Micro-PET Imaging

We randomly selected four mice from each group for the micro-PET imaging. Previously, mice were fasting and water deprivation for 6 h, the blood glucose levels were measured in normal range (7.0–10.0 mmol/L). In this experiment, mice were anesthetized with isoflurane (2% in 100% oxygen, 1 L/min) inhalation. 14.8–16.5 MBq of 18F-FDG tracer was injected to caudal vein when mice were fully anesthetized, micro-PET scanning performed 50 min after the injection. The experiment animal was placed in the prone position on the examination bed, with isoflurane continuously carried by breath mask to ensure animal remain completely unconscious throughout the 10 min scanning process, micro-PET captured single frame image. Image reconstruction: micro-CT scans were performed with an X-ray tube voltage of 80 kV, a current of 500 μA, an exposure time of 200 ms, filtered back projection (FBP) and CT photon attenuation correction were used for image reconstruction (pixel size 0.2 mm × 0.2 mm × 0.8 mm), micro-PET image frames were taken 30 s/frame. Regions of interest (ROIs) selection: experimenter (who is blind to the entire experiment process) manually selected three-dimensional ROIs of hippocampus on PET/CT images, in transverse, coronal and sagittal plane. The percentage of injected dose per gram of hippocampus tissue (%ID/g) was determined on micro-PET images. All images were traced by an investigator blinded to experimental design.

## Statistical Analysis

Statistical analysis was performed with software SPSS, version 17.0 (SPSS, Inc., Chicago, IL, USA), and data were expressed as mean ± standard deviation (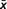 ± s). Hidden platform trial results were analyzed by repeated measures analysis of variance, while data of probe trial and micro-PET imaging were analyzed by single factor analysis of variance (one-way ANOVA), and LSD pairwise comparison method was used among groups. *P*-values less than 0.05 were considered statistically significant.

## Results

All mice were completed the Morris water maze test. Due to factors of anesthesia situation, tail vein injection and tracer’s metabolites, which may influence the scan results, we failed to get the data of one mouse in M group during the micro-PET imaging process. The data of this mouse were included in the analysis of Morris water maze results but excluded from the micro-PET data analysis due to its absence.

### Morris Water Maze Test

**FIGURE 2:**
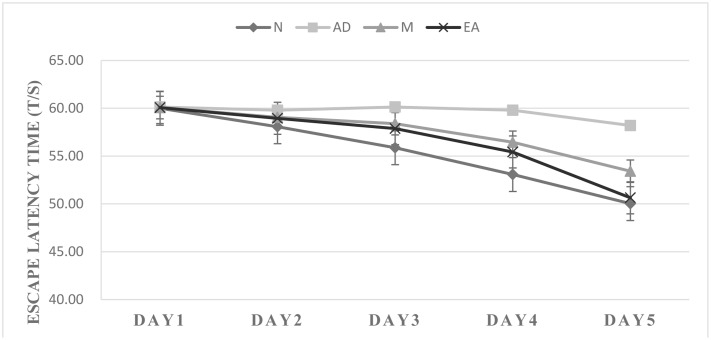
**Trends of escape latency time of each group in hidden platform trial**.

In the hidden platform trial, the escape latency time in each group showed a downward trend with the training time extension (**Figure 2**). Compared with the N group, longer escape latency time in AD group was observed (*P* < 0.01), suggesting that 7-month APP/PS1 mice shown an obvious disability in learning and memory. The escape latency time in the AD group was significantly longer than that in the M and EA groups (*P* < 0.01), and the escape latency time decreased more remarkably in EA group than that of the M group (*P* < 0.01) (**Table 2**).

**Table 2:** Comparison of escape latency time of each group in hidden platform trial (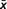 ± s, s, *n* = 10).

Groups	Cases	Day 1	Day 2	Day 3	Day 4	Day 5
N	10	60.00 ± 0	58.07 ± 0.74	55.87 ± 1.01	53.07 ± 1.76	50.03 ± 3.98
AD	10	60.00 ± 0	59.80 ± 0.71	60.13 ± 0.73 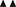	59.80 ± 0.71 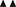	58.20 ± 3.22 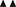
M	10	60.08 ± 0.50	59.06 ± 0.58	58.39 ± 1.08 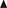 *	56.44 ± 0.91ˆ**	53.42 ± 2.55ˆ**
EA	10	60.07 ± 0.46	58.95 ± 0.58 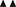	57.88 ± 0.80 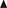 **	55.43 ± 0.86 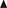 **	50.64 ± 2.21ˆ**

^
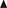
^*Compared with the N group, *P* < 0.05; ^
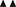
^Compared with the N group, *P* < 0.01; ^∗^Compared with the AD group, *P* < 0.05; ^∗∗^Compared with the AD group, *P*< 0.01*.

**FIGURE 3:**
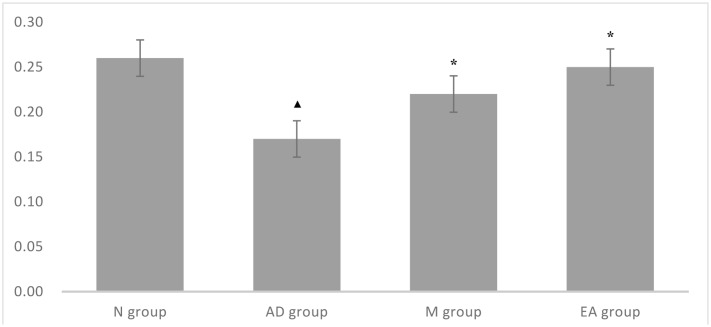
**Comparison of ratio of the swimming distance in platform quadrant to the total of each group in probe trial**. ^
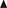
^Compared with the normal control group, *P* < 0.01; ^∗^ Compared with the model group, *P* < 0.01.

After 5-day training trail, probe trial was used for testing the maintenance of memory, while One-way ANOVA was performed to determine the ratio of the swimming distance in platform quadrant to the total swimming distance. A higher ratio of swimming distance in the platform quadrant could be considered as a higher level of memory retention. In this trial, we found that the ratio of AD group was significantly lower than those in N group (*P* < 0.01); both M and EA group showed a significant higher ratio than AD group (*P* < 0.01); and EA group occupied a higher percentage compared with the M group (**Table 3**; **Figure 3**).

**Table 3:** Ratio of the swimming distance in platform quadrant to the total of each group in probe trial (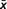 ± s, %, *n* = 10).

Groups	Cases	Ratio
lNormal Control Group (N)	10	0.26 ± 0.02
lModel Group (AD)	10	0.17 ± 0.02
lMedicine Group (M)	10	0.22 ± 0.02
lEA Group (EA)	10	0.25 ± 0.02

### Micro-PET Imaging of Hippocampus

**FIGURE 4:**
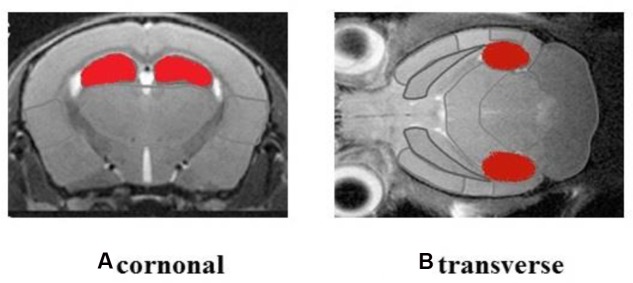
**Regions of interest (ROIs) of hippocampus were shown in red**.

**FIGURE 5:**
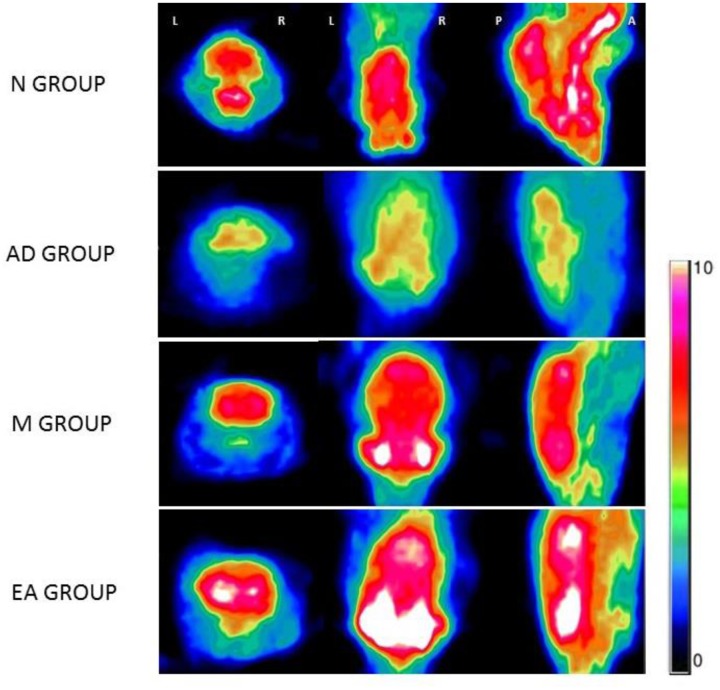
**Glucose metabolic rate of Each Group in micro-PET imaging**.

The ROIs of hippocampus for PET data analysis are outlined in **Figure 4**. Standard color code (from Minimum = 0 to Maximum = 10) was used to display the metabolic rate of glucose in mice hippocampus. The left of the observer presents the left of the animal. After the treatment, mice in M and EA groups showed an increased glucose metabolic rate than that in AD group (**Figure 5**). Based on data obtained from micro-PET scanning, we made further calculation and comparison of uptake rate of 18F- FDG per gram in hippocampus among groups. The results showed that the uptake rate in hippocampus of AD group was significantly lower than that in N, M and EA groups (*P* < 0.01). The uptake rate of EA group was higher than M group (**Table 4**).

**Table 4:** Comparison of uptake rate of 18F- FDG per gram in hippocampus of each group in micro-PET imaging (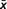 ± s, *n* = 4,4,3,4, %ID/g).

Groups	Cases	%ID/g
Normal Control Group (N)	4	7.75 ± 0.39
Model Group (AD)	4	3.88 ± 0.60 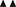
Medicine Group (M)	3	5.13 ± 1.27 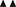
EA Group (EA)	4	6.35 ± 0.87 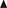 **

^
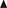
^*Compared with the N group, *P* < 0.05; ^
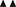
^Compared with the N group, *P* < 0.01; ^∗∗^Compared with the AD group, *P* < 0.01*.

## Discussion

Traditional Chinese medicine holds the opinion that “GV converged Yang channels” and “all Yang channels circulate in the head and go into the brain,” which indicates that GV could govern Yangqi over the body and closely related to brain functions. Any dysfunction of the brain might induce a series of mental, conscious and cognition diseases, such as AD. Based on the conventional theory that “Diseases at the point where a channel travels by can be treated by acupuncture on the channel.” In this experiment, GV20, GV29 and GV26, located on the pathway of the GV on the head, were selected for promoting the function of GV. A wealth of researches support that acupuncture treatment especially acupuncture at GV acupoints could have a positive effect on AD (Li et al., 2014; Zhou et al., 2015).

We chose APPswe/PS1dE9 double transgenic mouse as the AD model. This specific transgenic mouse is harboring chimeric mouse/human familial AD genes for β-amyloid precursor protein (APPswe) and a mutant human presinilin 1-246E (PS1-246E), which could propose a series of pathological changes of AD and commonly accepted in pathogenesis and prevention researches (Machová et al., 2008). One research found that active microglia around Aβ plaque increased significantly in 7-month-old APPswe/PS1dE9 mouse brain, while the level and function of transport proteins decreased with the increase of age (Chung et al., 2010). So far, some studies have shown that APPswe/PS1dE9 double transgenic mice demonstrated deficits in short-term memory, spatial memory loss and cognitive impairment at approximately 7–8 months of age and progressively throughout their lifespan (Gimbel et al., 2010; Scullion et al., 2011).

Here, we administered donepezil as positive control. Donepezil (Aricept) is an FDA-approved drug and the most commonly used anticholinesterase inhibitor for AD in clinical medication treatment, which has certain therapeutic effects in enhancing hippocampal neurogenesis and improving cognitive function. As an acetylcholinesterase inhibitor, donepezil extends its effects on depletion of nicotinic acetylcholine receptors and loss of cholinergic neurons. Due to its high selectivity, reversibility and sustained drug effects, donepezil was used in treat different stages of AD, from mild to severe. This effective drug is not only enhancing neurogenesis, improving cognition, learning and memory but preventing the aging progress. A number of studies using animal models have showed that donepezil possesses the neuroprotective activity *in vivo* and *in vitro* (Ryan et al., 2001; Takada et al., 2003), and clinical trials demonstrated that donepezil could considered as a safe and effective drug (Rogers and Friedhoff, 1996; Rogers et al., 1998; Bullock et al., 2005).

Alzheimer’s disease is characterized by an insidious onset and inexorable progression of brain atrophy, and common symptoms including gradual impaired memory, language obstacle, deterioration of visuospatial skill, count disturbance, behavioral and psychological abnormalities. So far, the criteria for clinical evaluation and assessment of therapeutic effect for AD remain unclear. At present, mini-mental state examination (MMSE) is used in screening test for cognitive function evaluate worldwide, but disadvantages such as low-sensitivity and influences by age, geographic and educational levels cannot be ignored. As advanced neural physiological activities, learning and memory related to multiple encephalic regions, a variety of intracellular signaling molecules and neurotransmitters are involved in such complex processes. Hippocampus is an important structure of brain’s limbic system and playing a crucial role in emotion, behavior, learning, and memory (Lamberty and Gower, 1993). In the 1960s, scientists studied memory and learning abilities on experimental animals and discovered that hippocampus played a vital role both in the short-term memory to long-term memory transition and spatial information distinguish processes. Morris primarily applied water maze device to detect the role of the hippocampus in 1984, and found that hippocampal damage has a serious negative impact on learning and memory function (Morris, 1984). Previous research has shown that hippocampal atrophy was known as an extremely sensitive and specific indicator of early AD, appeared unexpected in the pathological process (Jack et al., 1997). Rusinek proved that hippocampus was the only structure involved in early onset process of AD (de Leon et al., 1997). Imaging techniques such as MRI and PET have a major role in improving our understanding of the biology of AD. Laakso found that hippocampal atrophied significantly with its volume decreased in AD patients via MRI (Laakso et al., 2000). Recently, a meta-analyses research on the difference between mild AD and non-AD, found that using a variety of biomarkers and imaging examination in AD diagnosis, the sensitivity and specificity of PET imaging are 90 and 89% respectively, and non-AD subjects in control group showed 92% accuracy via PET/CT scanning (Bloudek et al., 2011). Therefore, the PET/CT may contribute to the differential diagnosis of AD.

Brain relies almost exclusively on glucose as its source of energy, and the glucose level is considered as an indicator of neuronal function. Many studies indicated that the pathogenesis of AD is directly associated with glucose metabolism disorders. Decreased intake of glucose, which may act as a neurodegenerative component of AD, is the direct substrate of cognitive impairment, and the rate of cognitive decline is driven by the rate of neurodegeneration. Some research suggests that metabolism diminution condition of brain existed before cognitive decline onset (Hoyer, 2004), and neuronal degeneration and loss could occur as a consequence of brain atrophy and reduced glucose metabolism conversely in AD patients (Nagren et al., 2010; Vallabhajosula, 2011; Braskie et al., 2012). Brain metabolism can be measured via fluorine-18 labeled fluorodeoxy glucose (18F-FDG) and PET imaging. To date, 18F-FDG is the most mature and widely accepted biomarker of brain metabolism in the diagnosis and efficacious assessment of AD. Studies proved that AD individuals showed gradually reduce in cortex glucose metabolism, which was positively correlated with the severity of AD symptoms (Politis et al., 2012; Dukart et al., 2013). Possessed the features of non-invasion, measurable and transferability from research to clinic, PET has emerged as a reliable and applicable method for both basic medical research and clinical practice in brain dysfunctions (Rowland and Cherry, 2008). We used micro-PET in this study, which has the 3R advantages (i.e., replacement of animals, reduction in the number used and refinement of techniques that alleviate potential pain and distress of the animals). CT images were acquired just before each PET scan to provide an anatomic reference for PET data.

In summary, the results of this study demonstrate that both donepezil and EA stimulations have positive efficacy on AD mice. EA shows better therapeutic effects on improving the spatial learning and memory ability, while also enhancing of glucose metabolism in hippocampus of APP/PS1 mice. Such therapeutic mechanism might closely relate to EA stimulates on GV, regulating qi and blood of the head, raising the lucid Yang, restoring consciousness and inducing resuscitation. These findings may indicate that EA could be a potential efficacious therapeutic technique for treating AD, and this research also serve as a stepping stone to future exploration in wide application of acupuncture.

## Ethics Statement

All experimental procedures were in accordance with the Guidelines for the Care and Use of Laboratory Animals of the Ministry of Science and Technology of the People’s Republic of China, and the Experimental Animal Research Ethics Committee of Beijing University of Chinese Medicine approved the study protocol.

## Author Contributions

JC: experimental design, data analysis and manuscript preparation; YT: data analysis and manuscript preparation; YL, KG, and XS: data collection and data analysis. All authors contributed to draft the manuscript and have read and approved the final manuscript; ZL: experimental design.

## Conflict of Interest Statement

The authors declare that the research was conducted in the absence of any commercial or financial relationships that could be construed as a potential conflict of interest.
